# On-Device Semi-Supervised Activity Detection: A New Privacy-Aware Personalized Health Monitoring Approach

**DOI:** 10.3390/s24144444

**Published:** 2024-07-09

**Authors:** Avirup Roy, Hrishikesh Dutta, Amit Kumar Bhuyan, Subir Biswas

**Affiliations:** Department of Electrical and Computer Engineering, Michigan State University, East Lansing, MI 48824, USA; royaviru@msu.edu (A.R.); duttahr1@msu.edu (H.D.); bhuyanam@msu.edu (A.K.B.)

**Keywords:** semi-supervised learning, privacy preserving, personalized machine learning, human activity detection, wearable devices, on-device learning, health monitoring

## Abstract

This paper presents an on-device semi-supervised human activity detection system that can learn and predict human activity patterns in real time. The clinical objective is to monitor and detect the unhealthy sedentary lifestyle of a user. The proposed semi-supervised learning (SSL) framework uses sparsely labelled user activity events acquired from Inertial Measurement Unit sensors installed as wearable devices. The proposed cluster-based learning model in this approach is trained with data from the same target user, thus preserving data privacy while providing personalized activity detection services. Two different cluster labelling strategies, namely, population-based and distance-based strategies, are employed to achieve the desired classification performance. The proposed system is shown to be highly accurate and computationally efficient for different algorithmic parameters, which is relevant in the context of limited computing resources on typical wearable devices. Extensive experimentation and simulation study have been conducted on multi-user human activity data from the public domain in order to analyze the trade-off between classification accuracy and computation complexity of the proposed learning paradigm with different algorithmic hyper-parameters. With 4.17 h of training time for 8000 activity episodes, the proposed SSL approach consumes at most 20 KB of CPU memory space, while providing a maximum accuracy of 90% and 100% classification rates.

## 1. Introduction

An increasingly sedentary lifestyle in modern societies has compounded many health problems such as diabetes, obesity, and high blood pressure. The high-resolution monitoring of daily activities and providing personalized feedback are found to be effective in reducing sedentary lifestyle. There are many commercially available wearable products such as the Apple Watch [1] and Fitbit watch [2] that can monitor and classify human activities using supervised machine learning methodologies. Such learning typically requires model training using pre-labelled data collected from many users. Once centrally trained, the model is loaded in those wearable devices before consumers can use them.

While this approach of model training works, the design cycle can be improved in the following two fronts. First, the activity data for supervised learning model training can potentially raise privacy concerns. This is because the activity data from a large population needs to be recorded for training. Inadvertent access to such personal fitness- and health-oriented data can be abused by insurance companies and organizations during job placement and hiring. Second, the model used in commercially available wearable is trained using data collected from a generalized population. This contrasts with model training in a subject-specific manner, which is known to provide better classification accuracies for activity monitoring applications.

This paper sets out to address these two design issues by employing a self-supervised activity classification approach in which activity data are recorded from the same subject for whom a classification model is trained iteratively. By not relying on data from others, it addresses the privacy concerns, and by training a semi-supervised model with the same person’s activity data, thus making it personalized. In the proposed approach, the entire learning process happens on-device, thus eliminating the needs for (i) transferring data over network links and (ii) storing and processing it on an out-of-device system. These further add to privacy preservation. The tradeoff is that the mechanism needs to be computationally and storage-wise light enough to be able to run on low-power embedded devices.

The on-device semi-supervised learning (SSL) [3] approach is specifically designed for learning with sparsely labelled datapoints. This light-weight mechanism is fully implemented in embedded wearable devices. The targeted application scenario is as follows: When a user starts using such a device, the semi-supervised learning algorithm in it starts “training itself” with the activity data collected from the device-integrated inertial measurement sensors (IMU) such as accelerometers. Based on pre-stored templates, if the algorithm can classify a current activity, it labels these data. As the device is used, this mechanism is used to label a small subset of all collected activity data. The self-supervised training and classification are performed simultaneously in an iterative manner. As the device is worn by the user for longer durations, the objective is to improve the classification performance over time. Special care is taken to reduce the computational and storage complexity of the iterative mechanism to keep it suitable for low-power wearable embedded devices. Furthermore, since training and data collection both happen on the same device, the target application setting ensures full privacy by not allowing the user data to ever leave the device. Personalization is achieved since the algorithm that classifies a user’s activities and is trained mainly using the data from the same user.

The main contributions of the paper are as follows First, it introduces a privacy-preserving and personalized semi-supervised learning mechanism that can perform human activity classification with accuracies that are comparable to neural network-based supervised learning. It also explores how this can be achieved with fewer labelled data and with low computational resources represented by computational and memory complexities. Second, two SSL variants, namely, population-based, and distance-based, are explored and evaluated for the application considered in the paper. Finally, all the above mechanisms are experimentally analyzed and evaluated from the standpoints of classifiability, classification accuracy, and computational/storage complexity. Accuracy figures are compared with those from benchmark supervised learning models.

## 2. Related Work

Human activity detection (HAD) in commercially available devices such as smartphones and smart watches is often performed with embedded sensors such as accelerometers and gyroscopes [4]. Different combinations of sensing modalities and their positions on the human body have been explored in the literature [5,6,7,8]. On the machine learning front, different forms of HAD systems have been realized using a variety of classifiers based on supervised learning [7,9]. The above works explore different elements of HAD systems like intraclass variability and interclass similarity. The challenges of disparity in relevant and irrelevant data have also been discussed.

To deal with person-specific activity types, which are often affected by individual physical attributes [10,11] and such, personalized human activity detection has been explored in [12,13,14,15,16,17,18,19,20,21]. While ref. [12] deals with it using biometry, refs. [13,14,15,16,17,18,19,20,21] deal with it at the classifier level. The works in [13,16] train supervised learning models using personalized data from the target user. Both of these works involve the transfer of the data collected from the target user to be transferred to an external server for supervised training. This method affects user privacy since the data are shared to an external device. Some relevant works in [17,18,19] consider transfer learning in a way of domain adaptation methods. Transfer learning has the convenience where explicit training data are not required from the target user device but has the disadvantage of the increased likelihood of selecting incorrect class data for training, thus degrading the overall classification performance. Refs. [20,21] present similar works which consider personalized learning models for classification tasks.

On-device learning is an effective solution for applications related to personalized applications. A relevant work in [22] uses similar pre-trained supervised learning models for on-device hydration-tracking. This hydration-tracking system also preserves the privacy of the target user data, but the usage of the supervised learning model requires a huge amount of training data. At times enough labelled data may not be available from the target user to train a supervised learning model. In this way, either privacy or personalization or both for a classifier model may be sacrificed, which becomes a point of major concern for these kinds of applications which require both privacy and personalization.

Semi-supervised learning (SSL) [3] is a possible solution for on-device learning in which classifiers can be trained on the device from a specific target user’s data, thus preserving the personalization feature of the model as discussed in [23]. SSL is also useful in this context because unlike in fully supervised learning, learning with SSL does not rely on the availability of extensive labelled data. It was shown in [24] that SSL models can potentially learn with sparsely labelled datapoints. This mechanism has proven to be effective for on-device applications involving limited computational power. A relevant work in [25] uses an SSL method based on the combination of deep learning and transfer learning. The usage of a deep learning model involves training using data from different users other than the target user. This affects the personalization feature of our premise in this paper. The work in [26] develops a human activity detection system using a bi-view semi-supervised learning to detect semantic human activities like having dinner, shopping, etc. This method also uses a windowed datapoint extraction technique and clustering mechanism as the basis of the classifier model, but not on the same device where the sensors are present. This method also involves a two-layered framework for the classification task, which becomes computationally expensive and is thus not very suitable for on-device self-training and classification.

The above works in the literature provide many definitive ideas for classifying human activities with or without using personalized classifiers. While semi-supervised learning has been proposed as an effective tool to train personalized classifiers of HAD, none of these works considers the feasibility of training on devices with computational constraints. Also, the aspect of computational complexity is absent in those approaches. The work in this paper addresses both these issue by developing a low-computation self-training mechanism using a semi-supervised learning algorithm. Both classification accuracy and computational complexity are considered as evaluation parameters. The different hyper-parameters and system parameters are analyzed based on their relevance for implementation on wearable devices with computational constraints.

## 3. System and Data Model

**Figure 1:**
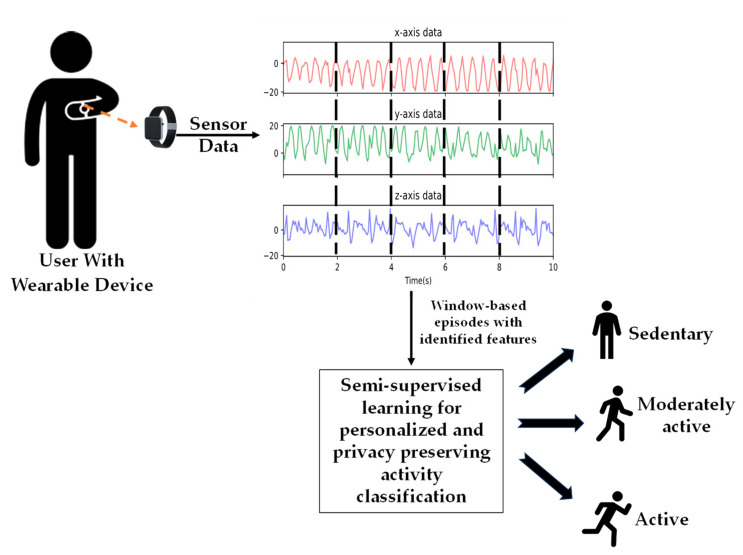
A wearable on-device human activity detection system.

This section outlines the adopted system and data model used by the proposed semi-supervised algorithms. Figure 1 depicts the entire system where the wearable device on the wrist of the user reads the human motion using embedded IMU sensors. The different classes of human activities are classified using the semi-supervised learning model self-trained using the motion data from the same user on the same wearable device.

### 3.1. Sensing Modality and Dataset

Activity classification is performed on data collected from a wearable embedded device containing Inertial Measurement Unit (IMU) sensors, storage, and a micro-controller. The IMU sensors capture user movements in terms of the acceleration of the wearable unit along three orthogonal axes. We have used a public-domain human activity dataset (i.e., Wireless Sensor Data Mining Lab-WISDM lab dataset from the University of California, Irvine [27]) generated from such a wearable unit. The dataset contains accelerometer time-series data collected from a smartwatch as 36 subjects perform six activities.

### 3.2. Segmentation and Feature Extraction

As the first processing step, the raw accelerometer data are pre-processed as follows: First, $l^{2}$ norm [28] is applied to standardize the data across all axes in order to ensure that the data are bounded within the range of −1 to +1. Subsequently, a window-based approach is used for segmenting data into 2 s long episodes. For each episode, two features are defined for each of the accelerometer axes. The first feature is the coefficient of variation, which reflects the acceleration variability across 40 samples spanning the 2 s long episode. The second feature is the number of mean crossing points within an episode. Physically, this one indicates the frequency of movements of the subject from its mean position. There may be a possibility of an exception occurring when there is no significant movement across the mean position in either of the three orthogonal directions. In order to handle this, ‘0’ has been set as the feature value for these special cases. Based on their physical implications, these two features are selected for their discriminatory abilities towards activity classification. Windowing and feature extraction from the raw time-series data helps in reducing the impact of noises in the raw data on the model. Windowing the time series into a fixed number of samples prevents the accumulation of errors which may happen in a continuous time series when the raw samples are provided to the model as inputs. Extracting features from consecutive time series’ samples means representing the set of samples by some feature values, which considers only the magnitude of significant movement by the subject in a particular direction during the 0.5 s episode duration. These feature values are fed to the learning model, which in turn helps in avoiding errors (if any) and any kind of model bias.

### 3.3. Class Definition

**Figure 2:**
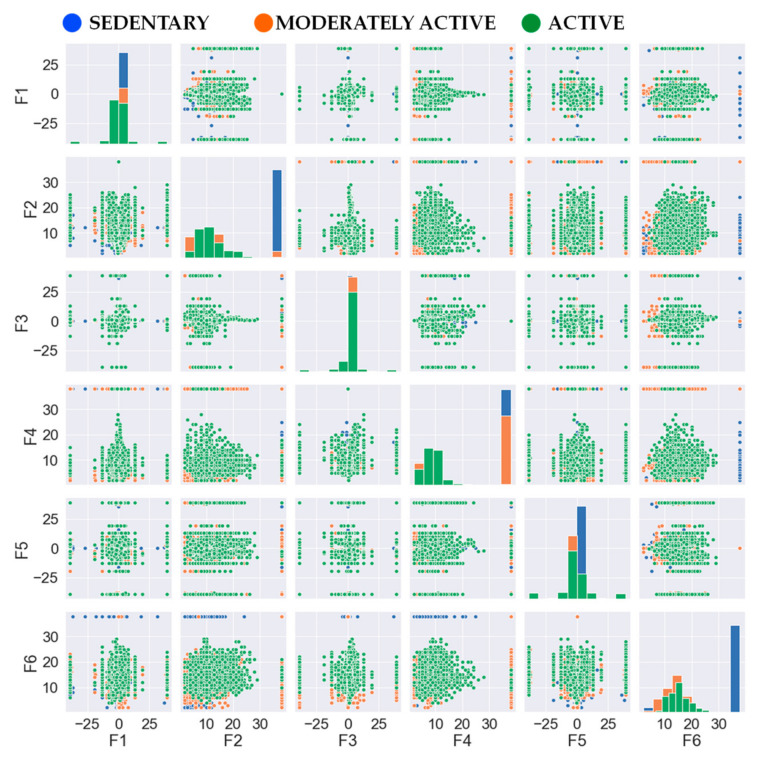
Feature distribution of the three classes.

Sedentary lifestyle has been attributed [29] as a main contributor to widespread obesity in modern urban societies in recent years. Monitoring and detecting sedentary lifestyles have been projected by the health research community as a useful tool to mitigate obesity and its many health effects. This has led researchers in recent years to actively investigate technology for classifying sedentary and non-sedentary activities [30,31]. Motivated by these works and the research trend, this paper sets out to deal with the problem of detecting sedentary activities but in a more personalized and computationally efficient manner. User activities are categorized into three distinct classes, namely, sedentary, moderately active, and active. The sedentary class encompasses stationary activities such as standing and sitting. Moderately active activities involve ambulatory motion, primarily represented by walking. Finally, the active class pertains to more vigorous physical activities, such as jogging. The correlation among all the features over the entire activity dataset is depicted in Figure 2.

### 3.4. Processing Pipeline

**Figure 3:**
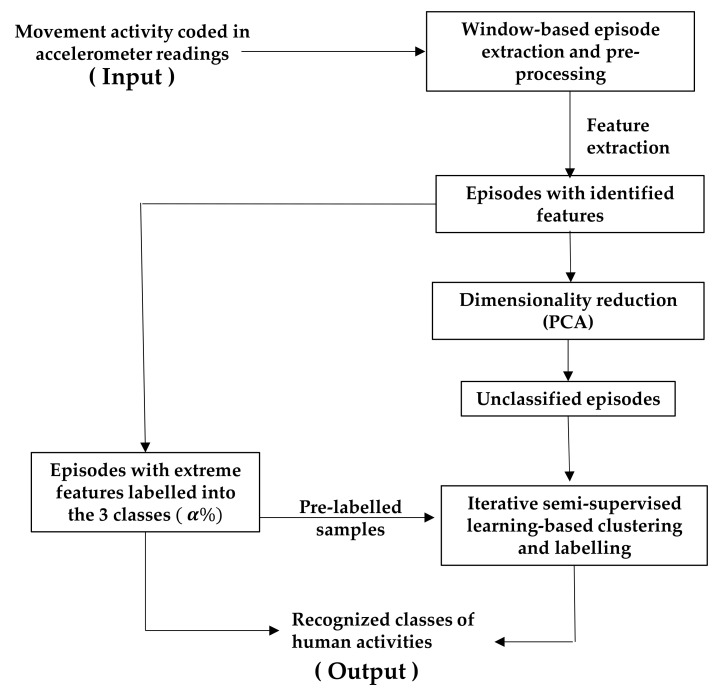
Pre-processing and classification pipeline.

Figure 3 depicts the entire processing dataflow for the proposed semi-supervised learning mechanism. It starts with acceleration sensing, with a sampling rate of 20 Hz. The resulting time series is then segmented into 2 s long episodes. The data in each window are subsequently normalized before extracting features as described above. The dimensions of the extracted features are then reduced for managing the computational complexity of processing. Such dimensionality reductions are achieved by choosing the top three principal components after applying principal component analysis (PCA) [32]. The proposed iterative semi-supervised learning (ISSL) is then applied in the presence of a small pool of pre-labelled data. Details of the ISSL process including adaptive data clustering and dynamic cluster-labelling are presented in the next section. This processing pipeline enables continuous improvement in activity prediction accuracy over time, thus providing a real-time mechanism to classify human activity with two-second temporal granularity.

## 4. Semi-Supervised Learning with Sparsely Labelled Datapoints

**Figure 4:**
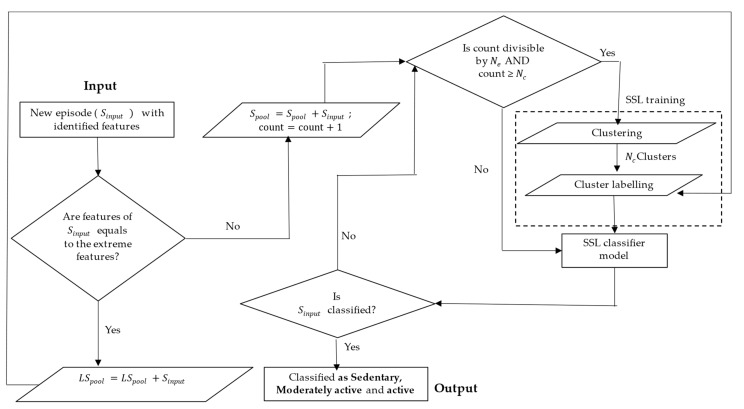
Iterative semi-supervised learning framework.

Figure 4 depicts the iterative semi-supervised learning framework which forms the basis of the classification model for human activities. The pre-processed accelerometer data arrives in the form of episodes of 2 s worth of data. The episodes are represented by the extracted features followed by a dimensionality reduction step. Reducing the dimensionality helps to reduce the computational load of the learning system, as described in Section 3. Each unlabelled incoming episode is added to a data pool ($S_{pool}$). A very small percentage (α) of incoming episodes which have extreme feature values beyond a threshold are pre-labelled with a designated activity class and added to a pre-labelled data pool ($LS_{pool}$). These are considered pre-labelled because they can be easily identified to be the parent of a class based on their extreme feature values matching to the corresponding class. These pre-labelled episodes are used in the SSL training phase for the cluster labelling step. The SSL training and activity prediction is performed on the unlabelled data pool ($S_{pool}$). The training is performed after every $N_{e}$ incoming episode. In the training step, first, all the existing episodes in the $S_{pool}$ are grouped into $N_{c}$ number of clusters based on their feature similarity in the clustering step. This clustering is performed by some popular clustering methods like k-means [33] or the Gaussian mixture model (GMM) [34]. There is the possibility of an exception when the number of datapoints in $S_{pool}$ **(count)** is less than the number of clusters ($N_{c}$). In those cases, there might be a run-time error during the clustering process. In order to handle this, a condition is placed where SSL training will only happen when the ‘count’ is greater than or equal to $N_{c}$. The $N_{c}$ clusters are labelled using the pre-labelled episodes ($LS_{pool}$), using any of the two cluster labelling methods: *population-based labelling* and *distance-based labelling*.

In the population-based labelling, the cluster is labelled on the basis of the highest number of pre-labelled episodes present in that cluster. For example, if cluster i has $M_{1}$ pre-labelled episodes from class A and $M_{2}$ pre-labelled episodes from class B, and if $M_{1}>M_{2}$, then cluster i gets labelled as class A. If cluster i does not have the highest number of pre-labelled episodes class of pre-labelled episodes or does not have a pre-labelled episode at all, it remains unlabeled. Thus, all the episodes in that cluster remain unclassified. This cluster labelling mechanism is shown in Algorithm 1.
**Algorithm 1:** Population-based cluster labelling1. **Input:** $LS_{pool}$, Cluster representatives (CR) of $N_{c}$ clusters.
2. **Output**:  Labels of $N_{c}$ clusters. 3. For $i=1\;to\;N_{c}$
4.    Calculate the number of pre-labelled samples in i-th cluster $M_{1}$, $M_{2}$,…,$M_{k},$ for k classes in i-th cluster. 5.    If max (M) exists:/*/max (*M*) is the unique class with the maximum number of pre-labelled episodes* 6.         Label cluster i with label of max(M)
7.      Else: 8.            Cluster i remains unlabeled. 9.        End If-Else 10.   End For

In distance-based labelling, the cluster is labelled based on label of the closest pre-labelled episode. For example, if cluster i has the minimum distance from the j-th pre-labelled episode which belongs to class A, then i is labelled class A. No clusters remain unlabeled in this variant of cluster labelling, until there are pre-labelled datapoints in the pre-labelled data pool ($LS_{pool}$). The clusters will remain unlabeled when there are no pre-labelled datapoints. This cluster labelling mechanism is shown in Algorithm 2.
**Algorithm 2:** Distance-based cluster labelling.1.  **Input:** $LS_{pool}$, Cluster representatives (CR) of $N_{c}$ clusters. 2. **Output**:  Labels of $N_{c}$ clusters. 3. For $i=1\;to\;N_{c}$
4.    Initialize the distance of cluster representative- i ($CR_{i})$ from pre-labelled samples as D;
5.    For $j=1\;to\;LS_{pool}$ /*/*$LS_{pool}$
*consists of the pre-labelled datapoints* 6.        Calculate distance of pre-labelled sample j from the $CR_{i}$ as $D_{j}$
7.    End For 8.    D(i) ← $min(D_{j})$*;//pre-labelled episode number with the minimum distance from ‘i’-th CR.* 9.    Label cluster ‘i’ with the label of ‘j’-th pre-labelled episode 10. End For

**Figure 5:**
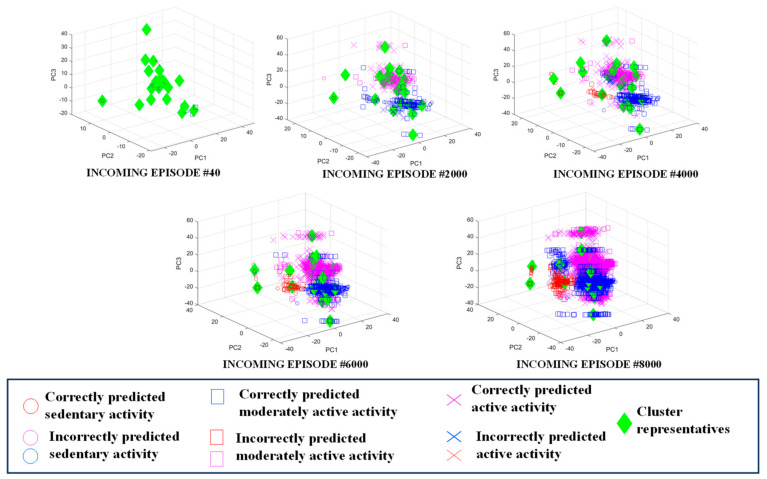
Example cluster model evolution with incoming episodes self-trained using iterative semi-supervised learning paradigm.

After the clusters are labelled using any one of the above two cluster labelling algorithms, the labelled clusters act as the trained classifier model. When a newly arrived and unseen episode comes in between training iterations, the episode is classified based on the most recently trained model, which are the labelled clusters. The new episode obtains the class label of the *nearest* cluster in the feature space. A few representative cluster models trained with incoming movement data from a user are shown in Figure 5. It can be seen how the model with 20 clusters expands and the classification accuracy improves with more incoming episodes. The quantitative performance of the classifier is formally presented in the next section.

*Personalization and Privacy:* The 2 seconds’ episodes represented by the extracted features are classified by the semi-supervised model embedded in a wearable device. The model is iteratively trained as more labelled episodes, which are very infrequent, come in. It also classifies unlabeled episodes in between those infrequent training episodes. Since both training and classification happen for the same user, the semi-supervised model acts in a very personalized manner. Since in this process no data need to be transferred outside the embedded wearable device, which performs both model training and classification, privacy of the user’s movement data is automatically preserved. This makes the approach personalized and privacy preserving at the same time.

## 5. Experiments and Results

An open-source human activity dataset (i.e., Wireless Sensor Data Mining Lab-WISDM lab dataset from the University of California, Irvine [27]) has been used for training and validation of the iterative semi-supervised learning model. Three broad classes of activities viz. *sedentary, moderately active and active* have been used from testing the proposed model as discussed in Section 3. The time-series data have been pre-processed and windowed into two-minute episodes. Six features, as described in Section 3, are extracted from each episode. Moreover, 8262 episodes (2754 from each class of activities) are used for the model evaluation experiments of the proposed iterative semi-supervised learning framework explained in Section 4.

The SSL model is trained using three principal components (i.e., using (PCA) [32]) extracted from the six features discussed earlier. Experiments involving different clustering algorithms, the hyper-parameter *number of clusters* ($N_{c}$), and system parameter α (i.e., *% of pre-labelled datapoints*) have been performed to evaluate the accuracy and complexity of the proposed SSL model. The accuracy metrics are *true positive (TP)*, *false positive (FP)*, *overall accuracy (Acc)*, and the *classification rate*, which is defined as the percent of classified datapoints among the actual number of datapoints. The computational complexity of the model is represented by the *computational time* and the *CPU memory usage* for each learning cycle. The accuracy and complexity are analyzed based on the performance of the SSL algorithm in order to train 8262 episodes (datapoints) for 100 runs. All the results involving the SSL algorithm are means of the results collected from 100 independent runs.

### 5.1. Pre-Trained Supervised Learning Model as a Benchmark

A pre-trained supervised model evaluated with a 10-fold cross-validation using the same dataset has been used as a benchmark for the proposed semi-supervised learning framework. The six extracted features mentioned in Section 3 have been directly used as the input to the NN model. The NN model is trained using the hyper-parameters mentioned in Table 1.

The mean performance (as mentioned in Table 2) of each accuracy parameter for all 10-fold cross-validation tests is used as a performance benchmark for the classification accuracy of the proposed model. It is observed that the NN model has mean true positives of 98.47%, 97.41%, and 98.19% for the three classes (i.e., sedentary, moderately active, and active), respectively. The mean overall accuracy is 98.03%.

### 5.2. Impacts of Feature Dimensionality Reduction on SSL

**Figure 6:**
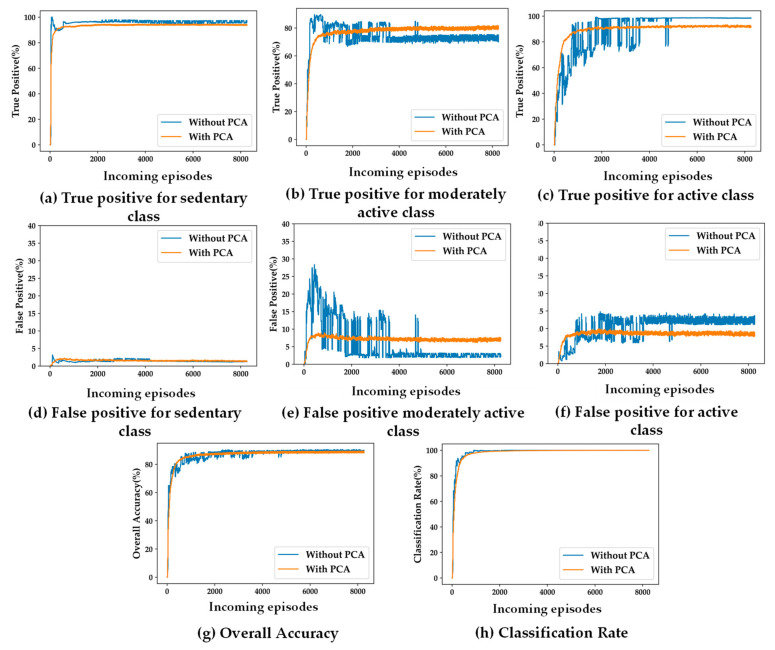
(**a**–**h**) Impacts of PCA on accuracy parameters for population-based SSL; (**a**–**c**): true positive for the three classes; (**d**–**f**) false positive for the three classes; (**g**) overall accuracy; (**h**) classification rate.

**Figure 7:**
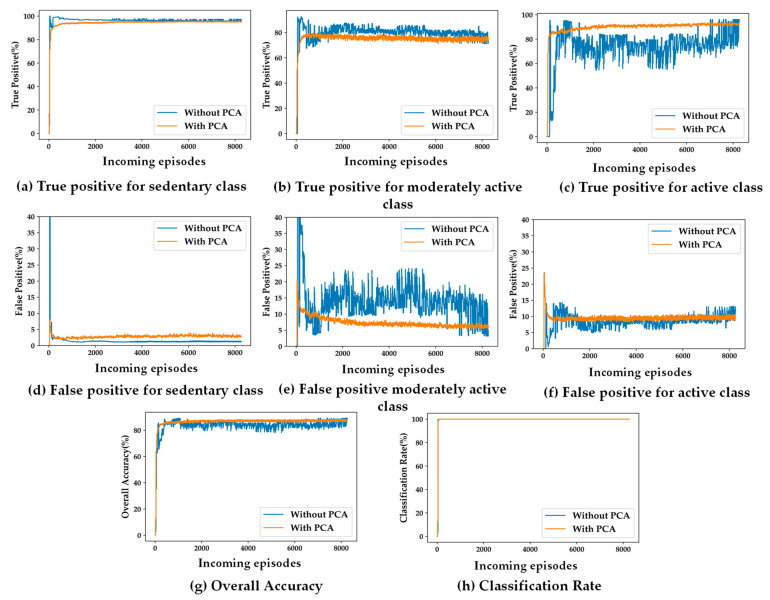
(**a**–**h**) Impacts of PCA on accuracy parameters for distance-based SSL; (**a**–**c**) true positive for the three classes; (**d**–**f**) false positive for the three classes; (**g**) overall accuracy; (**h**) classification rate.

The primary motivation of the proposed self-training algorithm is its suitability for the wearable system and its privacy-preserved personalized applications. Ideally, the semi-supervised learning-based self-training model needs to be computationally light due to the resource-constrained nature of the embedded wearable devices. To that end, PCA has been used to reduce the number of features from six to three. Figure 6 and Figure 7 depict the differences in accuracy parameters (TP, FP, Acc, and λ) for training the SSL model with and without using PCA, using the population-based and distance-based SSL algorithms, respectively. The results are presented for the two presented algorithm viz. population-based and distance-based. Moreover, 20 clusters formed by the k-means clustering algorithm are used for these experiments.

For both SSL algorithms, true positives (TP) and false positives (FP) for all the three classes are almost same for both the scenarios with and without using PCA. The overall accuracy (Acc) and the classification rate (λ) are also the same for both algorithms. The only observable difference is in the stability in TP, FP, and Acc performance with more incoming episodes. The performances with PCA have less oscillatory behavior after convergence compared to the case not using PCA. This makes it evident that a feature space with less dimensions makes the cluster models more definite in less time.

**Figure 8:**
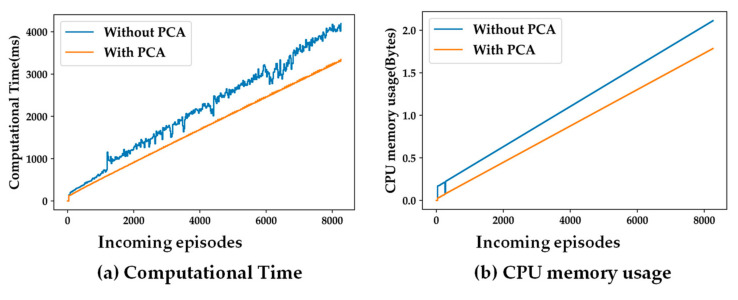
Impact of PCA on complexity for population-based SSL; (**a**) computational time; (**b**) CPU memory usage.

**Figure 9:**
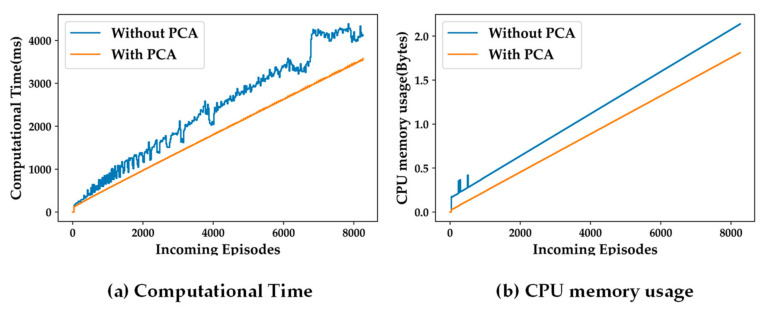
Impact of PCA on complexity for distance-based SSL; (**a**) computational time; (**b**) CPU memory usage.

Figure 8 and Figure 9 present the impacts of dimensionality reduction from the six original features to three principal components. It can be observed that more dimensions (features) of a data sample results in higher computational complexity. Thus, for both, the types of SSL algorithms, both computational time, and CPU memory usage go up when PCA is not used. Thus, the experiments analyzing the different hyper-parameters and system parameter of the SSL model are carried out using the three principal components obtained by using PCA on the original six features.

### 5.3. Impacts of Pre-Labelled Data Volume on SSL Performance

**Figure 10:**
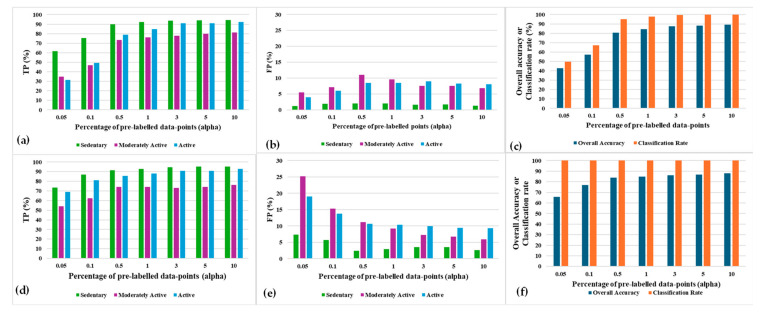
Post-convergence accuracy parameters’ results for (**a**–**c**) population-based SSL and (**d**–**f**) distance-based SSL, with varying α.

Figure 10 depicts the TP, FP, overall accuracy, and classification rate for SSL operating with different amounts of pre-labelled data, which is represented as α (in percentage). These experiments are performed with 20 clusters formed by the k-means clustering method in the SSL model. It can be observed that all the accuracy parameters converge faster and reach better performance values with increasing values of α. For the population-based SSL, lower α (i.e., 0.05%, 0.1%, 0.5%, and 1%) values are unable to achieve a 100% classification rate. The reasons are as follows: First, many clusters may the lack predominance of prelabelled datapoints from a particular class. Second, a cluster may not have any pre-labelled episodes altogether. All the datapoints in those clusters remain unlabeled and thus unclassified. Figure 10f shows that using the distance-based SSL, all the clusters and their datapoints are always labelled since this variant of SSL does not depend on the presence of any pre-labelled datapoints in a cluster. Rather, a cluster is labelled according to the label of the nearest pre-labelled datapoint in the distance-based SSL. The labelling in this mechanism is not impacted by whether the nearest pre-labelled datapoint is present inside or outside the cluster. Also, it can be observed that convergence is achieved faster in the distance-based SSL than in the population-based SSL.

**Figure 11:**
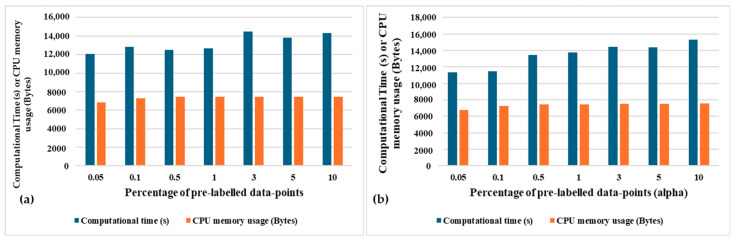
Total learning time and CPU memory usage for all the episodes using (**a**) population-based SSL and (**b**) distance-based SSL, with varying α.

Figure 11a,b depicts the total semi-supervised learning computational complexity for both variants of SSL. Complexity is represented by the total computational time in seconds and CPU memory usage in Bytes. It can be observed that the computational time and CPU memory usage are not dependent on the amount of pre-labelled datapoints for the population-based SSL since the number of pre-labelled datapoints vary in different clusters for different runs of the learning algorithm. On the other hand, it can be observed that more computational time is required for the distance-based SSL to learn 8262 episodes since the greater the number of pre-labelled datapoints, the greater the number of distance calculations required.

### 5.4. Impacts of the Number of Clusters ($N_{c}$) on SSL Performance

**Figure 12:**
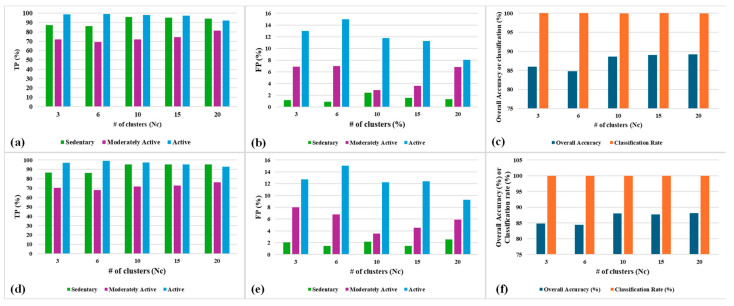
Post-convergence accuracy parameters’ results for (**a**–**c**) population-based SSL and (**d**–**f**) distance-based SSL, with varying *Nc*.

The classification in the proposed model occurs based on the clusters that actually contain datapoints based on their similarities. The number of clusters in the learning model is an important hyper-parameter which can determine the precision of classification as well as the computational complexity to train the cluster model. Figure 12 depicts the accuracy parameters after learning convergence. These results represent the accuracy parameters for both population-based and distance-based SSL algorithms with varying number of clusters in the model. These above results are obtained from experiments performed by setting the system parameter α to 10% and k-means as the clustering algorithm.

It can be observed that true positive (TP) values improve and false positive (FP) values decrease with higher $N_{c}$ for all three classes and for both the variants of SSL. As a result, the overall accuracy improves with a higher number of clusters ($N_{c}$). The overall accuracy performance does not improve much beyond 10 clusters for both population-based and distance-based SSL. Also, population-based SSL has marginally better accuracy (90%) compared to the distance-based SSL (88%). Both the variants of SSL reach a 100% classification rate, but distance-based SSL reaches this faster as all the clusters are labelled from the first cycle of learning as opposed to the population-based SSL.

**Figure 13:**
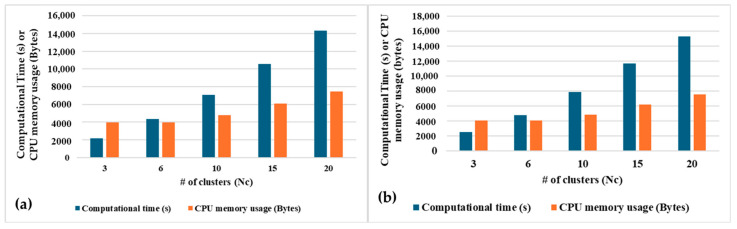
Total learning time and CPU memory usage for all the episodes using (**a**) population-based SSL and (**b**) distance-based SSL, with varying $N_{c}$.

In the Figure 13a,b, it can be observed that both variants of SSL become computationally expensive with a higher number of clusters. Total CPU memory usage required to train all the episodes of activities is the same for both population-based and distance-based SSL for each value of $N_{c}$. The total computational time required for iterative learning is marginally higher for the distance-based SSL compared to the population-based SSL. This is mainly because population-based SSL only counts the pre-labelled episodes in a cluster which is computationally less expensive than calculating the distance from each pre-labelled datapoint from a cluster center.

### 5.5. Impacts of Different Clustering Algorithms

Among the popular clustering methods, k-means (KM) and the Gaussian mixture model (GMM) are the best methods for the current approach. GMM has three useful variants based on the relevant covariance types: spherical (GMM_s), full (GMM_f), and diagonal (GMM_d). Based on the accuracy and complexity performances using the different clustering methods mentioned above, the best clustering method suited for the ideal SSL performance is analyzed.

**Figure 14:**
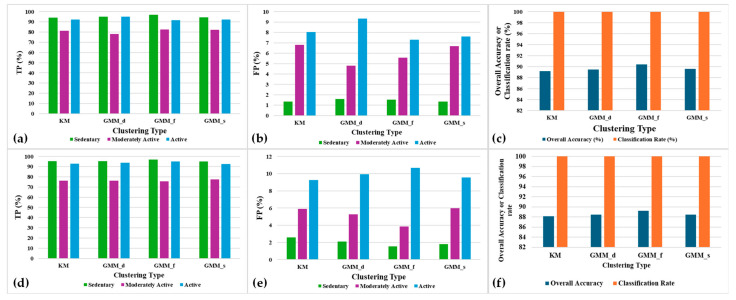
Post-convergence accuracy parameters’ results for (**a**–**c**) population-based SSL and (**d**–**f**) distance-based SSL for different clustering methods.

Figure 14a,b,d,e depict the TPs and FPs for all the three classes of activities along with the overall accuracy and classification rate for different clustering methods. The experiments are performed to provide 20 clusters ($N_{c}=20)$ using the different clustering methods, with α=10%. It turns out that GMM_f has the highest TPs and the least FPs for all the three classes. Subsequently, GMM_f has the best overall accuracy (Acc) for both the population-based and the distance-based SSL variants as shown in Figure 14c,f. This is because the full covariance type has each component with their own general covariance matrix, which makes the grouping of datapoints belonging to the same class of activities more precise. Population-based SSL has marginally better overall accuracy compared to distance-based SSL.

**Figure 15:**
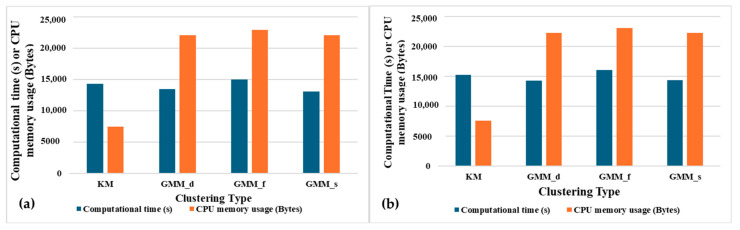
Total learning time and CPU memory usage for 8262 episodes using (**a**) population-based SSL and (**b**) distance-based SSL for different clustering methods.

Figure 15a,b show that GMM_f is computationally expensive with a very high total computational time and CPU memory usage for learning the entire dataset (8262 episodes). GMM_s has the second best overall accuracy after GMM_f with less computational time. But, the CPU memory usage is still very high. K-means requires much less memory usage compared to the GMM clustering types. Thus, when SSL is implemented on the wearable device with limited CPU memory, k-means should be preferred over GMM.

## 6. Discussion

This paper presents a fully on-device approach for human activity detection using a semi-supervised learning paradigm. The proposed SSL mechanisms self-trains in a fully personalized and privacy-preserving manner. The performance of the mechanism is compared with that of a pre-trained supervised learning model (NN) which forms a comparison benchmark. The post-convergence performance numbers for the benchmark and the different variants of the proposed SSL algorithm with the best algorithmic parameters (post-convergence) are summarized in Table 3. Since the NN model was pre-trained outside the wearable device, the computational time and CPU memory usage are not considered for the summarization purpose. Advantages of using an unsupervised dimensionality reduction technique prior to the SSL algorithm are also discussed in Section 5.2.

The proposed SSL model is analyzed and validated based on different learning hyper-parameters and system parameters. The results suggest that 5% or higher pre-labelled activity datapoints add precision to the model, therefore improving its classification performance. It has been also observed that using 20 clusters in the model with GMM full-type clustering yields an overall accuracy of 90%, with a 100% classification rate after learning convergence. However, since GMM_full is a computationally expensive method, a k-means clustering method is also explored for resource-limited scenarios. With 4.17 h of training time for 8000 activity episodes the k-means clustering-based SSL approach consumes at most 20 KB of CPU memory space while providing a maximum accuracy of 90% with a 100% classification rate. It is true that the SSL model cannot reach an accuracy (98%) as high as the pre-trained supervised model (NN) where the entire training dataset is pre-labelled. However, the accuracy of the SSL model (90%) with only 10% of pre-labelled datapoints is still comparable and within a practically acceptable range. These results indicate the feasibility of the proposed SSL paradigm for practical human activity detection using a personalized and privacy-preserving health monitoring system.

The proposed semi-supervised learning framework has the following limitations. It can be observed in Figure 8 and Figure 9 that the computational time and CPU memory usage keep increasing monotonically as the learning progresses with the accumulation of incoming episodes (datapoints) in the data pool. As the size of the data pool keeps increasing, more computational resources (time and CPU memory) are required. Thus, a necessary upgrade of the proposed framework is required such that the learning process should stop after the classification model has converged. Following the interest of the sedentary research community [29,30,31], this paper deals with three major relevant classes, namely, *sedentary*, *moderately active*, and *active*. The presented SSL framework can be scaled up to handle more activities beyond the sedentary-targeted clinical applications.

## 7. Conclusions

This paper presents an on-device semi-supervised human activity detection system that can learn and predict human activity patterns in real time. The proposed semi-supervised learning (SSL) framework uses sparsely labelled user activity events acquired from Inertial Measurement Unit (IMU) sensors installed as wearable devices. The objective is to learn classification in real-time. The proposed cluster-based learning model in this approach is trained with data from the same target user, thus preserving data privacy while providing personalized activity detection. Two different cluster labelling strategies, namely, population-based and distance-based, are employed to achieve the desired classification performance.

A comparative study of the proposed strategy has been presented in terms of classifiability and classification accuracies. Different accuracy parameters for each of the classes of human activities (true positive and false positive) as well as the overall accuracy of classifying all the classes of activities have been considered to evaluate the learning model formed by the proposed SSL framework. The proposed system is shown to be computationally efficient, which is relevant in the context of limited computing resources on typical wearable devices. The trade-off between classification accuracy and computation complexity is analyzed for different algorithmic hyper-parameters (number of clusters) and other system parameters (% of pre-labelled datapoints). Different clustering algorithms like k-means and the Gaussian mixture model (GMM) have also been experimented with as part of the framework evaluation. Computational time and CPU memory usage have been used as parameters to measure the time and space complexity of the proposed learning model for the different algorithmic parameters mentioned above. Extensive experimentation and simulation studies have been conducted on multi-user human activity data from the public domain to validate the proposed learning paradigm. It has been observed that the proposed semi-supervised online learning framework can achieve a maximum classification accuracy of around 90% with 10% pre-labelled as opposed to the pre-trained supervised learning model which achieves 98% classification accuracy with 100% pre-labelled datapoints.

## Figures and Tables

**Table 1:** Hyper-parameters for pre-trained neural network (supervised learning) model.

**# of input features**	6
**# of hidden layers**	1 (128 neurons)
**Activation function**	tanh (hidden layers), soft-max (output layer)
**Optimizer**	Adam
**Loss function**	Categorical cross entropy

**Table 2:** 10-fold validation results for a pre-trained neural network model.

Folds	Class 1: Sedentary		Class 2: Moderately Active		Class 3: Active		Overall Accuracy (%)
	True Positive (%)	False Positive (%)	True Positive (%)	False Positive (%)	True Positive (%)	False Positive (%)	
1	98.582422	0.7047926	97.174001	1.4925373	98.433106	0.7076425	98.06321453
2	98.207171	0.4873096	97.245849	1.3693113	99.022801	0.9037959	98.15736382
3	98.589847	0.7468712	96.593186	1.2952844	98.779992	0.9845288	97.98278644
4	98.907767	0.8259468	97.41205	1.4910336	98.073063	0.4853387	98.13071544
5	98.245614	0.5616851	98.436247	2.3472278	97.06945	0.2224469	97.91554599
6	98.371336	0.7831325	97.6	1.7828201	97.983871	0.4640839	97.98278644
7	98.479392	0.7899534	97.406807	1.6304348	98.217902	0.5234548	98.03657881
8	98.954984	1.2328213	96.847211	1.8540911	97.291835	0.362757	97.70037655
9	98.266828	0.5852674	97.243616	1.5496076	98.633441	0.7881973	98.0500269
10	98.103309	0.524405	98.217902	1.7112945	98.392929	0.4042854	98.23830016
Mean	98.470867	0.7242185	97.417687	1.6523643	98.189839	0.5846531	98.02576951

**Table 3:** Summary of post-convergence accuracy results and total computational complexity numbers for the pre-trained NN model (benchmark) and the different variants of semi-supervised learning model.

Learning Mechanism	Class 1: Sedentary		Class 2: Moderately Active		Class 3: Active		Overall Accuracy (%)	Classification Rate (%)	Total Computational Time to Train 8262 Episodes (s)	Total CPU Memory Usage to Train 8262 Episodes (Bytes)
	True Positive (%)	False Positive (%)	True Positive (%)	False Positive (%)	True Positive (%)	False Positive (%)				
Neural Network (Benchmark)	98.47	0.72	97.41	1.65	98.18	0.58	98.03	100	N/A	N/A
Population-based SSL (K-means, $N_{C}$ = 20, α = 10%)	94.19	1.33	81.21	6.81	92.17	8.03	89.18	99.17	14,308.92	7455.104
Distance-based SSL (K-means, $N_{C}$ = 20, α = 10%)	95.32	2.58	76.36	5.9	92.8	9.27	88.16	100	15,293.88	7559.6
Population-based SSL (GMM_d, $N_{C}$ = 20, α = 10%)	95.1	1.6	78.25	4.82	95.1	9.34	89.48	99.99	13,454.91	22,122.97
Distance-based SSL (GMM_d, $N_{C}$ = 20, α = 10%)	95.57	2.09	76.09	5.28	93.69	9.94	88.45	100	14,281.27	22,273.25
Population-based SSL (GMM_f, $N_{C}$ = 20, α = 10%)	97.04	1.53	82.4	5.58	91.72	7.3	90.38	99.99	15,003.91	22,930.06
Distance-based SSL (GMM_f, $N_{C}$ = 20, α = 10%)	96.93	1.55	75.7	3.86	95.14	10.69	89.255	100	16,059.36	23,080.42
Population-based SSL (GMM_s, $N_{C}$ = 20, α = 10%)	94.45	1.34	82.13	6.68	92.15	7.6	89.57	99.99	13,060.78858	22,117.19
Distance-based SSL (GMM_s, $N_{C}$ = 20, α = 10%)	95.16	1.79	77.6	5.96	92.622	9.55	88.46	100	14,382.66124	22,267.82

## Data Availability

An open-source human activity dataset (i.e., Wireless Sensor Data Mining Lab-WISDM lab dataset from the University of California, Irvine) has been used for the experiments of this research. The WISDM dataset can be found at https://archive.ics.uci.edu/dataset/507/wisdm+smartphone+and+smartwatch+activity+and+biometrics+dataset (accessed on 4 September 2023).
